# Late-Onset Ictal Asystole and Falls Related to Severe Coronary Artery Stenosis: A Case Report

**DOI:** 10.3389/fneur.2021.780564

**Published:** 2022-01-07

**Authors:** Francesco Fortunato, Angelo Labate, Michele Trimboli, Carmen Spaccarotella, Ciro Indolfi, Antonio Gambardella

**Affiliations:** ^1^Department of Medical and Surgical Sciences, Institute of Neurology, University Magna Graecia, Catanzaro, Italy; ^2^Cardiology Unit, Department of Medical and Surgical Sciences, University Magna Graecia, Catanzaro, Italy

**Keywords:** seizure, ictal asystole, SUDEP, ischemic heart, cardiac arrhythmia

## Abstract

**Introduction:** Ictal asystole (IA) is a rare, underestimated, and life-threatening cause of transient loss of consciousness and fall. Current treatment options for seizures associated with IA usually include cardiac pacemaker implantation. We report, for the first time, a case of IA that is related to coronary stenosis, which was resolved after coronary angioplasty.

**Case Presentation:** A 73-year-old man had a 2-year history of focal seizures with impaired awareness. Three months before our observation, he started to have sudden falls resulting in injury on several occasions. General and neurological examinations, as well as brain MRI, were unremarkable. Interictal electroencephalography (EEG) showed bitemporal spiking. Ictal video-polygraphy revealed a diffuse electrodecrement, followed by a buildup of rhythmic 4–6 Hz sharp activity, which was more evident in the left temporal region. After the seizure onset, the ECG showed sinus bradycardia, followed by sinus arrest that was associated with the patient's fall from the standing position. Afterwards, sinus rhythm returned spontaneously. A diagnosis of IA was made. A comprehensive cardiologic evaluation revealed a sub-occlusive stenosis of the left anterior descending artery. Successful coronary angioplasty resolved IA, levetiracetam was added, and no seizure or fall has occurred in the following 20 months. Moreover, he underwent a 7-day Holter ECG monitoring, and no asystole was depicted.

**Conclusion:** The present case was unique as it shows the potential association between IA and coronary stenosis, also suggesting a possible therapeutic role for coronary angioplasty. It also highlights the importance of carefully investigating epilepsy patients with falls, especially in the elderly, since IA-related falls can be easily misdiagnosed in older age. Thus, if IA is identified, a deeper cardiac evaluation should be considered. As seen in our patient, non-invasive diagnostic examination including routine, prolonged, and exercise ECG, as well as echocardiogram, were readily available and were informative in diagnosing cardiac abnormalities that are amenable to specific treatment.

## Introduction

Ictal asystole (IA) is a rare, underestimated, and life-threatening cause of transient loss of consciousness, which usually leads to traumatic falls with significant morbidity (1–3). An IA and a non-epileptic syncope may be difficult to distinguish if based solely on clinical grounds due to the similarity of the clinical features, as both are most likely the result of cerebral hypoperfusion (4, 5). Semiologic features supporting an epileptic origin include behavioral arrest and blank stare, preceding falls (4, 5). Also, anamnestic findings of automatisms, visual, olfactory, auditory or gustatory hallucinations, or the déjà vu sensations are indicative of a seizure origin (4, 5), as these symptoms are not observed with reflex faints. On the other hand, post-ictal confusion, a key distinguishing feature of IA, may be misdiagnosed with situational disorientation that occurs in some patients recovering from cardiac syncope (6). Likewise, some patients with focal epilepsy may have seizures presented with isolated syncope that may be challenging to diagnose (7). Moreover, the ECG evolution during an IA is largely indistinguishable from that of a vasovagal faint, with sinus tachycardia being followed by progressive heart rate slowing, which at times progresses to an asystolic pause (5). Consequently, it may be difficult to establish an unequivocal clinical distinction by clinical features or ECG alone (5). Therefore, the diagnosis of IA can be challenging unless the clinician suspects it, or if it is captured on video-polygraphic monitoring with the documentation of asystole, as produced by an ictal electroencephalography (EEG) discharge (1–3).

Most reported cases had late-onset IA on the ground of long-standing drug-resistant focal epilepsy, and IA typically appeared after the onset of epilepsy with delay of many years (1). Nonetheless, it has also been recognized as a new-onset IA group with high prevalence of pre-existing heart conditions (1, 8, 9). IA has been hypothesized as one of the several mechanisms of sudden unexpected death in epilepsy (10), and this risk highlights the need for complete seizure control, cardiac evaluation, and potential cardiac intervention (11). Indeed, current treatment options for seizures associated with IA include anti-seizure medications, epilepsy surgery, and cardiac pacemaker implantation (11), but no treatment guidelines currently exist. We report, for the first time, a case of IA related to coronary artery stenosis that was resolved after coronary angioplasty. We present the following case in accordance with the CARE reporting checklist. The study was approved by the local ethics committee and the patient gave the consent.

## Case Presentation

A 73-year-old man had a 2-year history of occasional episodes of impaired awareness and arrest of behavior, which lasted in <1 min. Three months before our observation, he started to have sudden falls resulting in injury on several occasions. He has never experienced chest pain or other cardiac symptoms.

**Figure 1:**
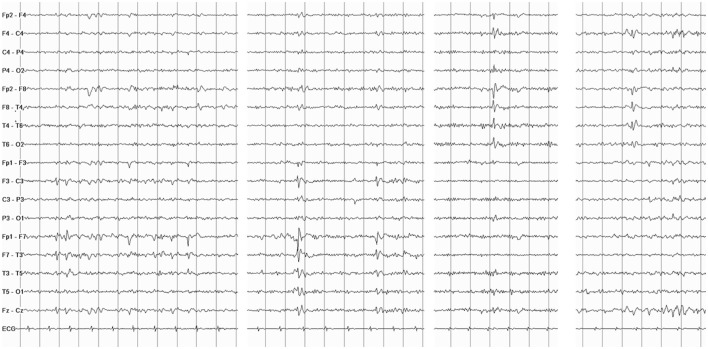
Interictal electroencephalography (EEG) shows spiking activity and slow sharp waves located over the temporal regions bilaterally.

**Figure 2:**
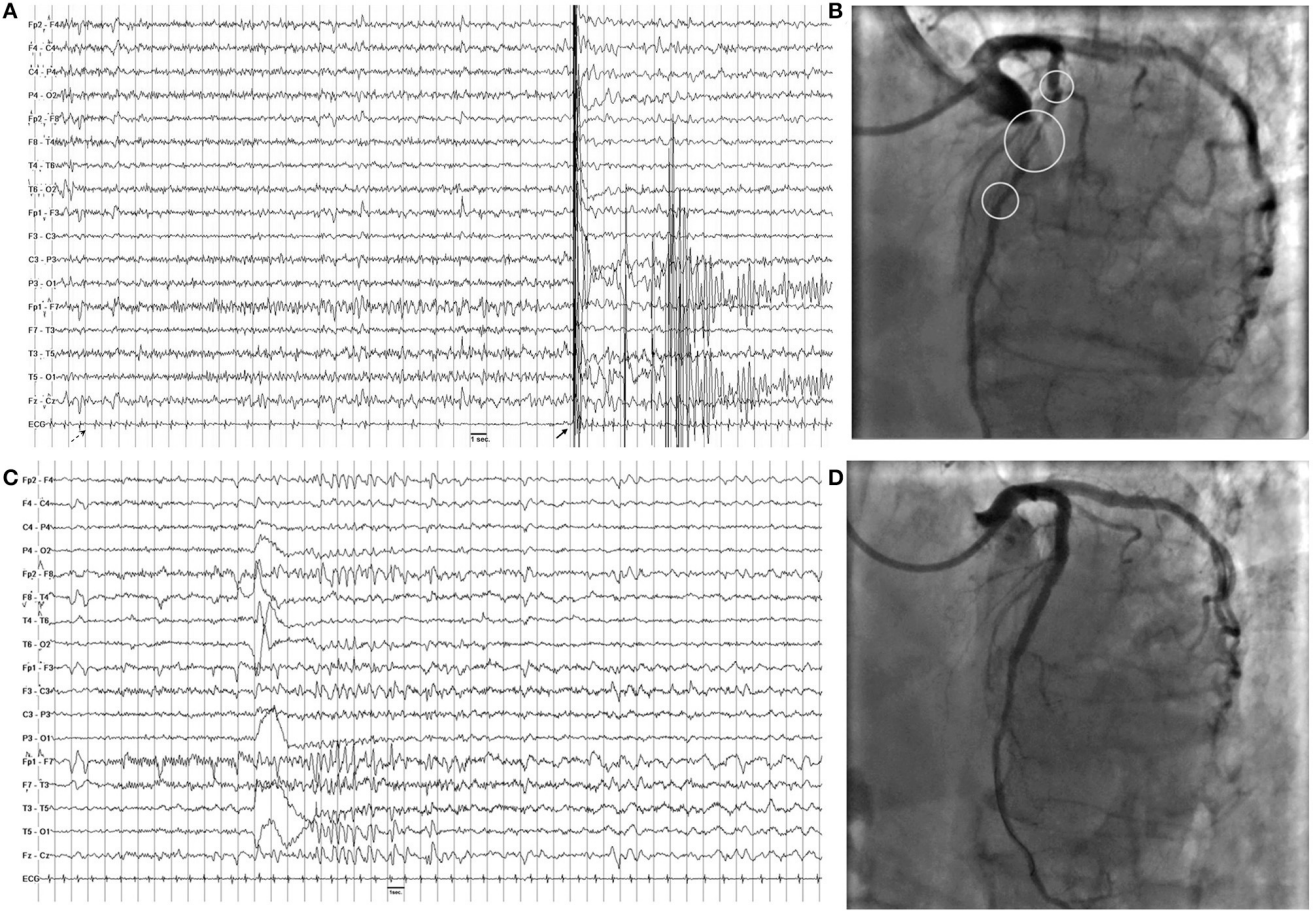
**(A)** Ictal asystole. On ictal EEG recording note the diffuse electrodecrement followed by buildup of rhythmic 4–6 Hz sharp activity more evident in the left temporal regions. On ECG, there is a progressive slowing of heart followed by asystole of 8 s. The dashed arrow indicates seizure onset, while the full arrow indicates the artifact due to the patient's fall from standing position. **(B)** Coronary angiography shows sub-occlusive stenotic traits (circles) of the left anterior descending coronary. **(C)** Ictal EEG after angioplasty shows a similar pattern with no modification of hearth rate on ECG channel. **(D)** Coronary angiography after angioplasty shows normal flow of the left anterior descending coronary.

General and neurological examinations, as well as brain MRI, were unremarkable. Interictal EEG showed sharp waves bilaterally located over the temporal regions (Figure 1). Video-polygraphic monitoring captured three typical epileptic seizures. The ictal EEG revealed a diffuse electrodecrement followed by buildup of rhythmic 4–6 Hz sharp activity more evident in the left temporal region (Figure 2). After seizure onset, the ECG always showed sinus bradycardia, followed on one occasion by eight-second sinus arrest that was associated with the patient's fall from the standing position (Figure 2). No fall occurred during the two ictal events with ictal bradycardia. Sinus rhythm returned spontaneously in all seizures. During the video-polygraphic monitoring, the episodes of asystole, and bradycardia coincided only with the ictal events. A diagnosis of ictal asystole (IA) (1) was made, and he underwent a thorough cardiologic investigation, in accordance to standardized protocol (12).

Electrocardiogram (ECG) recording and echocardiogram were unremarkable; tilt-test and cardiac enzymes were negative, while exercise ECG revealed ST elevation in V1, V2, and V4. Coronary angiogram showed sub-occlusive stenosis of the left anterior descending artery (Figure 2). Angioplasty with implantation of three everolimus-eluting stents resolved the coronary stenosis (Figure). In the following month, he continued to have staring spells, but no fall occurred. The Holter ECG monitoring result was normal. Interictal EEG continued to show bi-temporal spiking. During the follow-up video-polygraphic monitoring, three seizures were recorded, but none was associated with bradycardia or IA (Figure). Then, he started levetiracetam (1.0 g/day), and no seizure or fall, which was suspicious for seizure-induced cardiac asystole, has occurred in the following 20 months. Moreover, he underwent a 7-day Holter ECG monitoring, and no asystole was depicted.

## Discussion

Ictal asystole (IA) has been considered a rare complication of epileptic seizures, affecting <1% of patients with refractory seizures who underwent video-EEG monitoring (9). The IA seems to be a multifactorial condition and different predisposing factors seem to contribute to the ictal manifestation depending on whether it appears at the beginning of the epilepsy or later (6). In the new-onset IA, female gender and a preexisting heart condition may serve as predispositions in an otherwise mild epilepsy, while in late-onset IA, neuronal network changes in a chronic and therapy-resistant epilepsy may contribute to the ictal manifestation (6). Growing evidence also indicates that IAs are more frequent than was previously recognized and represent an important cause of falling (1, 2, 10). It is also likely that IA may be more common in the elderly, since epilepsy and seizure-induced falls are common in older adults, as well as cardiac and autonomic changes that are more frequently seen in old age (13). In addition, IA-related falling can be easily misdiagnosed in the elderly, since falls occur in more than one-third of the older population. Furthermore, not all seizures in the same patient give rise to IA. A systematic review quantified the short-term recurrence risk of IA to about 40%, suggesting also that in case of clinically suspected IA the recording of one or two seizures is not sufficient to rule out IA (14). Moreover, as seen in our patient, those with IA and longer asystole duration were considered “high risk patients,” since asystole duration >6 s is strongly associated with ictal syncope and falls (15). Therefore, a correct diagnosis is of paramount importance in such patients, as aggressive seizure control and cardiac pacemaker implantation are advocated to prevent serious injuries from falls related to IA. In this way, the present case was unique as IA and sudden falls were resolved after successful coronary angioplasty. Thus, awareness of this previously unrecognized triggering relationship has important implications for treatment and prognosis.

In our patient, it might be questioned whether coronary artery disease and IA are causally related. We believe that the interaction of the ictal temporal discharge superimposed on a chronic coronary is probably responsible for the genesis of ictal asystole in our patient. There is now a large body of evidence of frequent peri-ictal cardiac dysfunction and stress in association with different seizure types (8, 10, 12, 13). Indeed, a seizure activity may have deleterious impact on the structural integrity of the heart and its vasculature leading to myocardial fibrosis, accelerated atherosclerosis, systolic and diastolic dysfunction, and arrhythmias. Likewise, coronary artery diseases, especially stenosis of left anterior descending artery, are risk factors for almost any kind of arrhythmia, and contribute to ictal heart rate abnormalities, altered autonomic function, and potentially deleterious arrhythmias (16, 17), which may be a pre-requisite for sudden unexpected death in epilepsy (8, 10, 12, 13). During our patient's first video-EEG/ECG monitoring prior to angioplasty, all recorded seizures were always associated with bradyarrhythmia or asystole. Conversely, no change of heart rate occurred during seizures was recorded after coronary angioplasty. Moreover, at longer follow-up, the patient has no further experience of sudden fall, which is suspicious for seizure-induced cardiac asystole, as the 7-day ambulatory ECG monitoring has ruled out any asystole. Thus, it is conceivable that the damaged myocardium related to severe coronary disease greatly contributed to asystole during seizures in our patient. Obviously, further studies with longer follow-up and preferably long-term cardiac monitoring with a loop recorder are warranted to strengthen this relationship between IA and cardiac disease.

It is well-known that ictal epileptic discharges can cause changes in cardiac rhythm: they are more frequently associated with ictal tachycardia, while more rarely connected with ictal bradycardia or IA (18). Both ictal bradyarrhythmia and IA mostly coincide with focal seizures with impaired awareness and are predominantly seen in temporal lobe epilepsy (1, 13, 19, 20). Also, our patient had focal seizures of temporal origin, and video-EEG monitoring was a valuable tool for diagnosing seizure-induced cardiac asystole, as well as for guiding subsequent therapy. Our patient has EEG recordings that demonstrated diagnostic focal changes in the temporal regions at seizure onset, with the characteristic semiologic features of unresponsiveness and of staring, then, after about 15 s, short periods of sinus bradycardia followed in, on occasion by asystole, with a duration of 8 s.

Experimental evidence points to an early involvement of sympathetic and/or parasympathetic discharges in triggering the ictal cardiac changes, but the autonomic alterations in epilepsy are complex, and so are the mechanisms and interactions that support them. The IA is considered as primarily a seizure-driven phenomenon: cortical centers comprising the central autonomic network thought to be primarily responsible for triggering a “vagal storm” in ictal-induced bradycardia and IA (5). The hypothesis is that IA might be a direct consequence of epileptic activity involving cortical and subcortical structures, especially amygdala nucleus, cingulate gyrus, insula cortex, thalamus, and/or hypothalamus, all linked to the central regulation of cardiovascular functions (17).

Furthermore, many patients manifest both ictal bradycardia or tachycardia at various times in conjunction of their seizures (5). Many intriguing hypotheses have been proposed to explain the “tachycardia-bradycardia dichotomy” in epilepsy (17, 19). Moreover, the background cardiovascular status of patients with epilepsy is expected to influence the cardiac responses during seizures (9, 16). As far as we are aware, in a systematic review collecting 157 cases of IA, it was found a 28% of preexisting heart conditions in the new onset IA group, compared to 8.5% in the late-onset IA group. (9). Thus, we can speculate that in our case, the hearth rate changes were primarily driven by the focal seizures, but coronary artery disease could also contribute, shifting the “tachycardia-bradycardia dichotomy” toward the bradycardia and IA.

Finally, it is convincible that the prevalence of IA or ictal bradycardia is currently underestimated (17–19). Accordingly, data from out-patient cohorts have illustrated that bradyarrhythmias may occur at a higher frequency than what was reported from video-electroencephalogram recording centers (18). In a recent study, we also depicted ECG abnormalities, most frequently bradyarrhythmia, in ~15% of patients with a confirmed diagnosis of refractory epilepsy (12). More importantly, some patients have potentially showed to have life-threatening cardiac arrhythmias, which may make them more vulnerable to seizures that may become fatal (8).

## Conclusions

The present case was unique as it shows the potential association between IA and coronary stenosis, also suggesting a possible therapeutic role for coronary angioplasty. It also highlights the importance of carefully investigating falling as associated with patients with epilepsy (20) especially in the elderly, since IA-related falls can be easily misdiagnosed in older age. Thus, if IA is identified as such, a deeper cardiac evaluation should be considered. As seen in our patient, non-invasive diagnostic examinations including routine, prolonged and exercised ECG, as well as echocardiogram, are readily available and informative in diagnosing cardiac abnormalities that are amenable to specific treatment.

## Data Availability Statement

The original contributions presented in the study are included in the article/supplementary material, further inquiries can be directed to the corresponding author/s.

## Ethics Statement

The studies involving human participants were reviewed and approved by Comitato Etico Sezione Area Centro, Calabria, Italy. The patients/participants provided their written informed consent to participate in this study. Written informed consent was obtained from the individual(s) for the publication of any potentially identifiable images or data included in this article.

## Author Contributions

FF, AL, and AG contributed to conception and design of the study. FF wrote the first draft of the manuscript. MT, CS, and CI had a major role in the acquisition, analysis, and interpretation of the data. AG and AL revised the manuscript critically for important intellectual contents. All authors contributed to manuscript review, revision, and approved the submitted version.

## Conflict of Interest

The authors declare that the research was conducted in the absence of any commercial or financial relationships that could be construed as a potential conflict of interest.

## Publisher's Note

All claims expressed in this article are solely those of the authors and do not necessarily represent those of their affiliated organizations, or those of the publisher, the editors and the reviewers. Any product that may be evaluated in this article, or claim that may be made by its manufacturer, is not guaranteed or endorsed by the publisher.

## Abbreviations

ECG: electrocardiogram
MRI: magnet resonance imaging.
